# The Sweet Taste of Adapting to the Desert: Fructan Metabolism in *Agave* Species

**DOI:** 10.3389/fpls.2020.00324

**Published:** 2020-03-24

**Authors:** Arely V. Pérez-López, June Simpson

**Affiliations:** Department of Genetic Engineering, Cinvestav Unidad Irapuato, Guanajuato, Mexico

**Keywords:** Agavaceae, agavins, signaling, metabolism, adaptation

## Abstract

Over 70% of *Agave* species, (159 of 206) are found in Mexico and are well adapted to survive under hot, arid conditions, often in marginal terrain, due to a unique combination of morphological and physiological attributes. In the pre-Columbian era agaves were also key to human adaptation to desert terrain. In contrast to other species such as cacti or resurrection plants, *Agaves* store carbohydrates in the form of fructan polymers rather than starch or sucrose, however, properties specific to fructans such as a strong hydration shell, the ability to be transported through phloem, variable composition throughout the *Agave* life-cycle and accumulation in succulent tissues and flowers suggest a potential for multiple functional roles. This mini-review summarizes current knowledge of molecular and biochemical aspects of fructan metabolism in *Agave* species.

## Introduction

**FIGURE 1:**
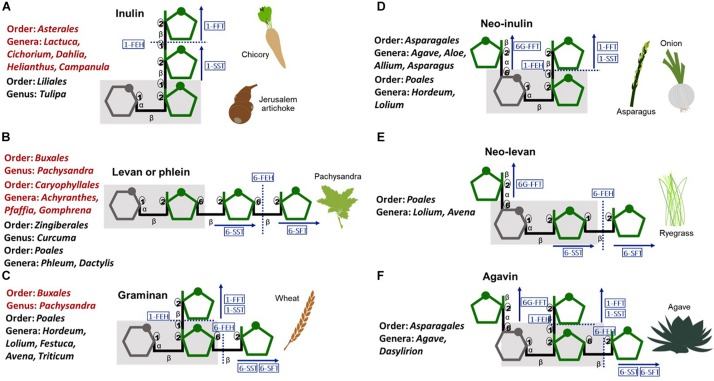
Schematic representation of plant fructans, their structural diversity and the enzymes involved in their metabolism. **(A)** linear inulin and **(B)** levan, **(C)** branched graminan, **(D)** neo-inulin, **(E)** neo-levan, and **(F)** highly branched agavin. Gray-glucose, green-fructose, gray shadow-sucrose moiety. Blue rectangles-enzymes:1-SST-sucrose:sucrose1-fructosyltransferase, 1-FFT-fructan:fructan1-fructosyltransferase, 6-SFT-sucrose:fructan 6-fructosyltransferase, 6G-FFT-fructan:fructan 6Gfructosyltransferase, FEH-fructan exohydrolase. Red text-dicotyledons, Black text-monocotyledons.

Fructan polymers, are synthesized by some bacteria and fungi and an estimated 15% of angiosperms including both monocotyledons and dicotyledons from different genera (Hendry, 1993; Figure 1). In plants, fructan polymers are described based on their structure and complexity (Versluys et al., 2018). Although neo type fructans have only been described in monocotyledons, no strong correlation exists between the type of fructan polymers and the genus or species in which they occur, supporting independent evolution of fructan metabolism.

Fructans are water soluble, flexible fructose based polymers synthesized from sucrose and accumulating in the vacuole. They can act as a long-term reserve carbohydrate in some plant species, alone or in combination with starch.

Fructans are an alternative to starch for long-term carbohydrate storage. Starch, composed of linear amylose or branched amylopectin glucose (hexose) polymers, accumulates in chloroplasts, whereas fructans produced by adding fructose monomers to sucrose are stored in vacuoles. Fructans are structurally flexible, highly soluble, accumulate to high levels, and have the ability to associate with cell membranes (Van den Ende, 2013). These properties are intrinsic to their roles in response to stress (Versluys et al., 2018) or developmental signals (Bolouri Moghaddam and Van den Ende, 2013). Fructans are exploited commercially as a replacement for sugar or fats, as fiber or prebiotics (Vijn and Smeekens, 1999) and have useful properties for drug delivery and cryoprotection (Audouy et al., 2011; Gupta et al., 2019).

*Agaves* evolved during the Miocene period and synthesis and storage of fructans was an important factor in adaptation to drier environments (Arakaki et al., 2011). *Agave* species range from the Canadian/United States border to the Northern region of South America (Gentry, 1982; Garcia, 2007). Whereas some species such as *A. deserti* or *A. americana* are adapted to wide temperature ranges others such as *A. tequilana* will not thrive at temperatures below −4°C or above 36°C (Nobel et al., 1998), demonstrating that tolerance mechanisms are complex.

Artificial selection of *Agaves* mainly took place in Mexico where 58% of species are endemic (Gentry, 1982; Garcia, 2007). Pre-Columbian cultures exploited these plants for food, fiber, construction and beverages and they were essential elements of nomadic life styles. *Agave* fructans provide the raw material for production of tequila and mescal, are being developed as components of treatments for diabetes and obesity (Franco-Robles et al., 2019) and as a resource for low-cost, carbon neutral production of bioenergy (Niechayev et al., 2019).

## *Agave* Fructans

The presence of fructans in *Agave* species was first recorded by Ekstrand and Johanson in 1888 as cited by Suzuki and Chatterton, 1993. In common with other members of the order Asparagales, *Agave* species synthesis inulin and neo series fructan polymers (Figure 1) and a new class of neofructans (subsequently known as “agavins”) was first identified in *A. tequilana* (Mancilla-Margalli and Lopez, 2006). Agavins are the most complex plant fructans described to date, comprising neoseries type fructans elongated at all three possible linkages (Figure 1). The composition of the fructan pool in *A. tequilana* varies as plants age, with agavins increasing in abundance in relation to inulins (Mellado-Mojica and Lopez, 2012).

In *Agave* leaves starch accumulation is largely limited to stomatal guard cells with minimal accumulation in other leaf cells (Zavala-Garcia et al., 2018). The presence of oligofructans containing 3–5 fructan residues (3–5 degrees of polymerization, D.P.) in *Agave* leaves indicates that sucrose produced by photosynthesis is metabolized to produce fructans rather than starch (Wang and Nobel, 1998) have shown that these oligofructans can accumulate in vascular tissue and are transported through the phloem. Although the transport mechanism is unknown, it is most plausibly by polymer trapping (Zhang and Turgeon, 2018). However, the presence of fructans in the extracellular space (apoplast) (Raveh et al., 1998) and putative roles in defense, signaling and membrane protection indicate that an apoplastic mechanism cannot be ruled out.

**FIGURE 2:**
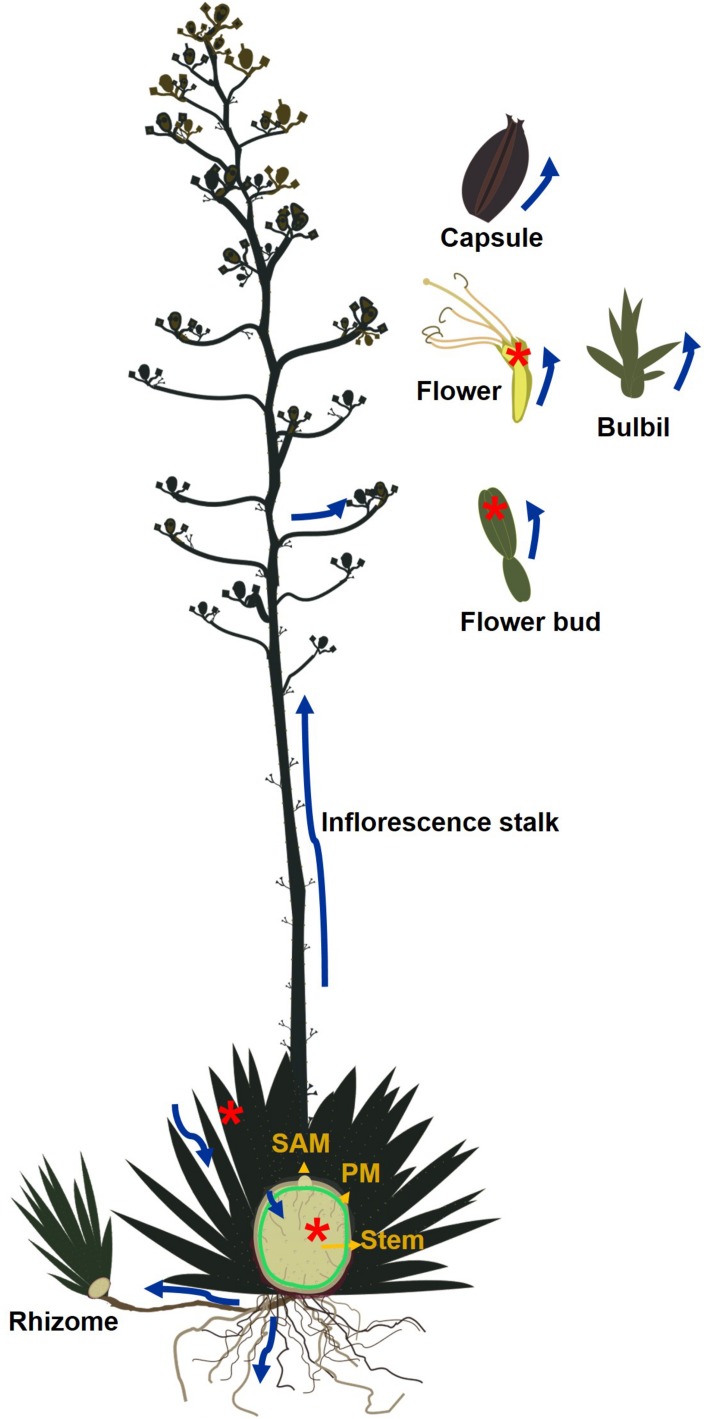
Fructan and/or sucrose mobility throughout an *Agave* plant. Blue arrows indicate fructan movement, green circle indicates the starch layer and peripheral meristem. Red asterisks indicate tissues where agavins are synthesized. SAM-shoot apical meristem. PM-Peripheral meristem.

Oligofructans and/or sucrose transported from leaves are either metabolized to starch that accumulates in the peripheral meristem region between the leaf base and the stem (Zavala-Garcia et al., 2018; Figure 2) or converted to complex fructans for long-term storage in the vacuoles of stem tissue (Mellado-Mojica et al., 2017).

## Fructan Metabolism in *Agave* Species

To synthesize agavins and inulins 4 fructosyltransferase (FT) activities (1-SST, 1-FFT, 6-SFT, and 6G-FFT, Figure 1) are needed whereas degradation of fructans is carried out by fructan exohydrolases (FEH) that may be specific for β 1→2 or β 1→6 linkages or act on both. FT and FEH in common with vacuolar and cell wall invertases are members of Plant Glycoside Hydrolase Family 32 (PGHF32). The first *Agave* FT to be characterized was a 1-SST from *A. tequilana* (Avila-Fernandez et al., 2007) and by RNAseq 15 members of PGHF32 from *A. tequilana, A. deserti, and A. victoriae-reginae* were later identified (Avila De Dios et al., 2015). Sequence based predictions of enzyme activities have also been confirmed for some *A. tequilana* enzymes using the *P. pastoris* system (Cortes-Romero et al., 2012). cDNAs encoding 6-SFT or 1-FFT type enzymes have not yet been conclusively identified perhaps due to low or tissue specific expression. Alternatively, some *Agave* FT enzymes may have multiple activities as reported for a 6G-FFT from onion (Weyens et al., 2004). *In silico* modeling supports this hypothesis since (Huang et al., 2018) have shown that the predicted structure of an *A. tequilana* 6G-FFT differs from those identified in *A. deserti* and *A. sisalana.*

*In silico* expression patterns for genes encoding invertases and FEH across three different *Agave* species (*A. tequilana*, *A. striata* and A. *victoriae-reginae*) are consistent, whereas FT expression is highly variable (Avila De Dios et al., 2015). For example isoforms encoding 1-SST enzymes from *A. tequilana* and *A. striata* showed similar tissue specific patterns whereas those identified for *A. victoriae-reginae* varied widely and 6G-FFT encoding genes of *A. victoriae-reginae* and *A. striata* are strongly expressed in vegetative tissue in contrast to *A. tequilana*. Expression patterns for all three *Agave* species showed high levels of expression for both FT and FEH in floral tissue (Avila De Dios et al., 2015) suggesting that fructans are not only being degraded but are also being synthesized in these organs.

Transcriptome analysis of the vegetative to reproductive transition in *A. tequilana* revealed no differential expression for starch metabolism related genes (Zavala-Garcia et al., 2018) whereas fructan related genes are highly expressed in SAM tissue in comparison to leaf tissue. In particular a 6G-FFT isoform is specifically and strongly expressed at the initial stage of the reproductive phase (Avila De Dios et al., 2019).

## Biological Functions of *Agave* Fructans

In *Agave* species fructans provide a source of carbohydrates for the vegetative to reproductive transition. Inflorescences can grow at a rate of 4–10 cms per day to reach 10 m or more (Valenzuela, 2003) and produce thousands of flowers, capsules, and seeds (Escobar-Guzman et al., 2008). Under cultivation, inflorescences are removed to avoid depletion of fructan reserves. Delgado Sandoval et al., 2012, showed that as the reproductive stage initiates, development of photosynthetically active leaves is suppressed and the SAM differentiates. Genes encoding FEH and invertases increase their expression during bolting (Avila De Dios et al., 2019) and leaves and stems senesce indicating that carbohydrate reserves are being harnessed for flowering.

Fructan reserves are also exploited during asexual reproduction since suckers produced from rhizomes or bulbils produced on inflorescences (Figure 2) also benefit from carbohydrates stored in the mother plant and may not survive if detached too early (Szarek et al., 1996). To accomplish these functions fructans must be mobilized over significant distances. Active fructan metabolism in floral tissue suggests carbohydrate availability could be limited by the rate of turnover or transport. Fructans may act as precursors to nectar production in floral tissue since *A. palmeri* produces 74 mg of nectar/flower composed mainly of glucose and fructose (Riffell et al., 2008). Alternatively fructans may be involved in generating osmolarity fluxes that lead to flower opening as described for *Campanula rapunculoides* (Vergauwen et al., 2000).

*Agaves* are perennial, monocarpic species with life cycles of 5 to over 50 years. They remain unresponsive to cues such as photoperiod or temperature, which induce flowering in annual or polycarpic species and probably respond to age-determined signals involving carbohydrate regulation. It could be speculated that accumulation of specific agavins produced by the 6G-FFT isoform described above may serve as age related molecular signals (Salinas et al., 2016) have also shown that neofructan levels increase in drought stressed *A. barbadensis* suggesting that neofructans play important functional roles.

The natural habitat of *Agave* species is in marginal desert terrain. Localization of fructans in hydrenchyma tissue in succulent *A. victoria-reginae* leaves (Singh et al., 2020) supports the evolution of fructan accumulation as an adaptation of Agavaceae to arid conditions. Consistent with these observations (Morales-Hernandez et al., 2019) showed that *Agave* fructans have a higher hydration shell in comparison to inulin and have predicted bioprotectant properties equivalent to trehalose. Suarez-Gonzalez et al., 2014 have shown that *A. tequilana* and *A. inaequidens* respond to cold and elicitors by increased FT expression and fructan production, consistent with roles in stress tolerance mechanisms.

## Discussion and Perspectives

Biochemical analysis has shown the presence of fructans in all organs of different *Agave* species and the quantity and complexity of these polymers varies depending on specific tissue and plant age. The monocarpic, perennial life cycle, large genome size and lack of molecular tools for *Agave* species have hampered molecular/genetic analysis, however, transcriptome data has allowed preliminary classification, and characterization of cDNAs and enzymes involved in fructan metabolism. The failure to identify 2 key enzymes may reflect low or transient gene expression or multiple enzyme activities. Functional genetic analysis in *Agave* is inefficient but heterologous systems such as *A. thaliana* and *P. pastoris* are being exploited and development of a genome sequence will resolve questions regarding isoforms, gene structure and regulatory elements. Comparisons of fructan metabolism on an evolutionary level between related taxa such as *Yucca* and *Aloe spp.* and aspects of coevolution with nectar feeding pollinators will also be feasible. Sub-cellular localization of FT or FEH enzymes, detailed gene expression patterns and aspects of fructan mobility must also be addressed to provide insights to roles in signaling and stress tolerance.

*Agaves* represent an invaluable resource in relation to development of agricultural systems on marginal land with resilience to climate change. However, indiscriminate collection of wild plants leads to decimation of natural populations and their pollinators. Fructans are the basis for the commercial exploitation of *Agaves*, therefore, understanding *Agave* fructan metabolism, its multiple roles in the *Agave* life-cycle and in adaptation to different habitats will facilitate strategies for exploitation and conservation. The current challenge in Mexico is how to exploit *Agave* fructans under a profitable, sustainable and socially pertinent agricultural system.

## Author Contributions

JS developed the outline of the manuscript. JS and AP-L wrote the manuscript and designed the figures. AP-L prepared the figures.

## Conflict of Interest

The authors declare that the research was conducted in the absence of any commercial or financial relationships that could be construed as a potential conflict of interest.
