# Corrigendum: The Sweet Taste of Adapting to the Desert: Fructan Metabolism in *Agave* Species

**DOI:** 10.3389/fpls.2020.00659

**Published:** 2020-05-28

**Authors:** Arely V. Pérez-López, June Simpson

**Affiliations:** Department of Genetic Engineering, Cinvestav Unidad Irapuato, Guanajuato, Mexico

**Keywords:** Agavaceae, agavins, signaling, metabolism, adaptation

In the original article, there was a mistake in the legend of **Figure 1**. The correct legend appears below.

“**Figure 1**. Schematic representation of plant fructans, their structural diversity and the enzymes involved in their metabolism. **(A)** linear inulin and **(B)** levan, **(C)** branched graminan, **(D)** neo-inulin, **(E)** neo-levan and **(F)** highly branched agavin. Gray-glucose, green-fructose, gray shadow-sucrose moiety. Blue rectangles-enzymes:1-SST-sucrose:sucrose1-fructosyltransferase, 1-FFT-fructan:fructan1-fructosyltransferase, 6-SFT-sucrose:fructan 6-fructosyltransferase, 6G-FFT-fructan:fructan 6G fructosyltransferase, FEH-fructan exohydrolase. Red text-dicotyledons, Black text-monocotyledons.”

In addition, the error in the legend of **Figure 1** was carried over into the text as the word “pentose” should have been removed. A correction has been made to the **Introduction**, paragraph 3:

“Fructans are an alternative to starch for long-term carbohydrate storage. Starch, composed of linear amylose or branched amylopectin glucose (hexose) polymers, accumulates in chloroplasts, whereas fructans produced by adding fructose monomers to sucrose are stored in vacuoles. Fructans are structurally flexible, highly soluble, accumulate to high levels, and have the ability to associate with cell membranes (Van den Ende, 2013). These properties are intrinsic to their roles in response to stress (Versluys et al., 2018) or developmental signals (Bolouri Moghaddam and Van den Ende, 2013). Fructans are exploited commercially as a replacement for sugar or fats, as fiber or prebiotics (Vijn and Smeekens, 1999) and have useful properties for drug delivery and cryoprotection (Audouy et al., 2011; Gupta et al., 2019).”

The authors apologize for this error and state that this does not change the scientific conclusions of the article in any way. The original article has been updated.
